# Novel Pilot Curriculum for International Education of Lymphoma Management Using E-Contouring

**DOI:** 10.1200/JGO.2016.008755

**Published:** 2018-01-11

**Authors:** Raymond B. Mailhot Vega, Omar F. Ishaq, Inaya Ahmed, Luis Rene, Beatriz E. Amendola, Kenneth S. Hu

**Affiliations:** **Raymond B. Mailhot Vega**, **Omar F. Ishaq**, **Inaya Ahmed**, and **Kenneth S. Hu**, Laura and Isaac Perlmutter Cancer Center, New York University School of Medicine, New York, NY; **Luis Rene**, Centro de Radioterapia, Rosario, Argentina; and **Beatriz E. Amendola**, Innovative Cancer Institute, Miami, FL.

## Abstract

**Purpose:**

The International Lymphoma Radiation Oncology Group (ILROG) published consensus guidelines on the management of Hodgkin disease (HD) and nodal non-Hodgkin lymphoma (NHL), which became the most downloaded articles from *International Journal of Radiation Oncology, Biology, and Physics*. E-contouring workshops allow for interactive didactic sessions, allowing participants to see case-based contouring in real time. A pilot 1-hour curriculum was developed with the objective of reviewing ILROG guidelines for HD and NHL management with incorporation of e-contouring tools. This represents the first international education intervention in Spanish using e-contouring with a pre- and postintervention questionnaire.

**Methods:**

A 1-hour presentation was prepared in Spanish reviewing the ILROG recommendations for HD and NHL. The review was followed by the author’s demonstration of contour creation using patients with HD and NHL prepared for the American Society for Radiation Oncology’s 2015 e-contouring lymphoma session. A five- question evaluation was prepared and administered before and after intervention. A two-tailed paired *t* test was performed to evaluate any significant change in test value before and after intervention.

**Results:**

A total of nine quizzes were collected before and after the intervention. The average test score before the intervention was 75.6%, and the average test score after the intervention was 86.7% (*P* = .051). Four students scored 100% on both the pre- and postintervention evaluations, and no student had a decrease in score from pre- to postintervention evaluation. The topic with the lowest score tested dose consideration.

**Conclusion:**

A substantial but nonsignificant improvement in test evaluation was seen with this pilot curriculum. This pilot intervention identified obstacles for truly interactive didactic sessions that, when addressed, can lead to fully developed interactive didactic sessions.

## INTRODUCTION

The International Lymphoma Radiation Oncology Group (ILROG) published consensus guidelines on the management of Hodgkin disease (HD) and nodal non-Hodgkin lymphoma (NHL), which became the most downloaded articles from *International Journal of Radiation Oncology, Biology, and Physics*.^1,2^ The guidelines provide a thorough and comprehensive set of recommendations including treatment volume principles, dose considerations, and planning techniques.

The consensus guidelines are particularly valuable because the field of radiation management of lymphoma is evolving, particularly in regard to target and field design. Historically, extended-field radiation therapy (EFRT) had been implemented as the norm for lymphoma treatment. With a reduction in field size, involved-field radiation therapy (IFRT) replaced EFRT as a result of its reduced toxicity because of the reduction in irradiated tissue. As positron emission tomography (PET) imaging has supplemented the oncologic workup of patients with lymphoma, IFRT is now giving way to involved-node radiation therapy (INRT) and involved-site radiation therapy (ISRT).

With an evolving field, practitioners must stay knowledgeable and feel comfortable if they choose to evolve their treatments, particularly if the benefit is a reduction in patient toxicity and improved quality of life. Medical education sessions, workshops, and guidelines all represent avenues practitioners can take to stay informed as management paradigms shift. One such education intervention involves e-contouring sessions, which allow participants to contour didactic cases and compare their results to the contoured volumes of an expert.^3^ These sessions are also interactive and allow for participants to ask questions of the session leader. An e-contouring workshop for lymphoma is provided by the American Society for Radiation Oncology (ASTRO), providing case-based learning for practitioners.

Although ASTRO provides e-contouring sessions for its participants, its audience is predominantly American, and its content is delivered in English. The advancement of lymphoma field design from IFRT to INRT and ISRT is not restricted to the United States, particularly as PET use expands globally. A novel, pilot, 1-hour curriculum was developed in Spanish for presentation at the 2015 meeting of the Asociación Latinoamericana de Terapia Radiante Oncológica (ALATRO) with the objective of reviewing ILROG guidelines for HD and NHL management with incorporation of e-contouring tools as part of the ASTRO International Education Subcommittee E-Contouring Ambassador Program. This represents the first collaborative lymphoma e-contouring program between ASTRO and ALATRO, before which there have been collaborations for other malignancy sites. This is the first program coupled with a metric to evaluate learning with a pre- and postintervention questionnaire.

## METHODS

A 1-hour presentation was prepared in Spanish reviewing the ILROG recommendations for HD and NHL for presentation at the 2015 ALATRO conference in Rosario, Argentina. The material informing the lecture and education session was obtained from the following two sources: the series of ILROG recommendations published in *International Journal of Radiation Oncology, Biology, and Physics* and the lymphoma e-contouring cases presented at the 2015 national ASTRO conference.^1,2^ The presentation was delivered using Microsoft PowerPoint (Microsoft, Redmond, WA). The education session reviewed the creation and contouring of gross tumor volume, clinical target volume, internal target volume, and planning target volume as recommended by ILROG; history of field design; dose selection; ISRT; INRT; the distinction between primary and consolidative management of HD and NHL; radiation therapy management in recurrent HD and NHL; and the use of PET in radiation therapy planning. The presentation curriculum also reviewed the methodology and results of the following three landmark randomized controlled trials: HD10, the British National Lymphoma Investigation, and FORT (4 Gy Versus 24 Gy Radiotherapy for Patients With Indolent Lymphoma).^4-6^ In addition to the aforementioned didactics, the session incorporated cases to consolidate participant learning apart from the e-contouring review that followed the presentation. The cases included in lecture format were stage IA diffuse large B-cell lymphoma (DLBCL), stage IIA DLBCL, stage IIAX DLBCL, and stage IA follicular lymphoma.

**Fig 1:**
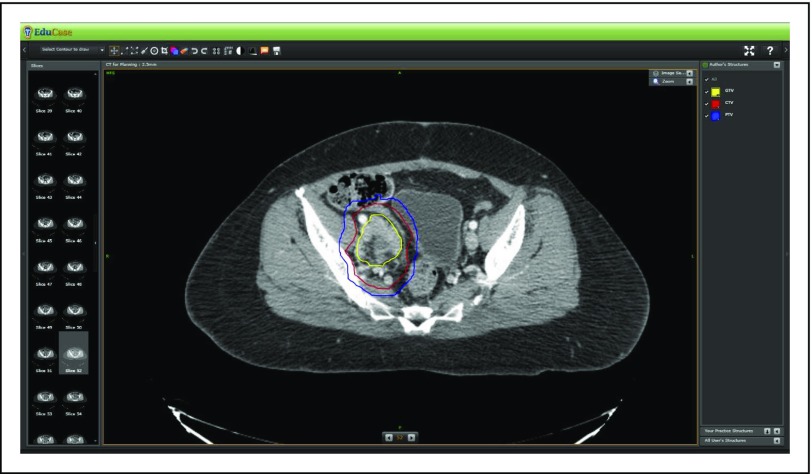
A sample axial slice of a case patient with stage IA nodular lymphocyte-predominant Hodgkin lymphoma of the inguinal region from the e-contouring platform EduCase. CTV, clinical target volume; GTV, gross tumor volume; PTV, planning target volume.

The review was followed by demonstration of contour creation using HD and NHL patient cases prepared for ASTRO’s 2015 e-contouring lymphoma session using the platform of EduCase from RadOnc eLearning Center (Fremont, CA). These patient cases spanned histologies and circumstances of presentation. A patient with stage IA nodular lymphocyte-predominant Hodgkin lymphoma after right inguinal excision was presented, as was a patient with stage IIAX classic Hodgkin lymphoma. The latter patient case also included a demonstration in the utility of deep inspiratory breath hold for the ability to spare cardiac structures. A representative axial slice from the e-contouring workshop from the patient with stage IA nodular lymphocyte-predominant Hodgkin lymphoma is shown in Figure 1.

The intervention was performed under the operation of the ASTRO domestic ambassador e-contouring program. In such exchange programs, it is ideal that participants are provided with access to an e-contouring Web site and instructional video before intervention, as well as a postintervention opportunity to contour, as is performed at ASTRO and European Society for Radiotherapy and Oncology (ESTRO) e-contouring sessions. For this intervention, such ideal implementation was not feasible at the 2015 ALATRO meeting because attendees did not have individual computer access and Internet access was unavailable. To carry out demonstration of the lymphoma volumes in the aforementioned patient cases, the presenter saved the e-contouring platform data offline a priori to allow for presentation and demonstration of lymphoma management. Volumes were reviewed axially with the attendees.

### Statistics

Before the education session, the didactic material was reviewed by the authors, from which a learning assessment was prepared. Ultimately, a five-question learning evaluation was created. The learning assessment was administered at two separate occasions—once before the intervention and once after the intervention. Test scores before and after intervention were treated as continuous variables, and comparative statistical evaluations were undertaken to evaluate whether a significant change in score occurred after the educational intervention. A two-tailed paired *t* test was performed to evaluate any significant change in test value before and after intervention. The α was set at .05. All statistical analyses were conducted using Microsoft Excel.

## RESULTS

**Fig 2:**
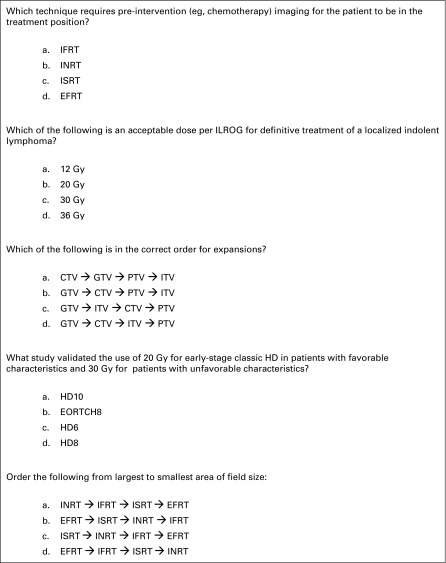
An English version of the five-question educational assessment. CTV, clinical target volume; EFRT, extended-field radiation therapy; EORTC, European Organisation for Research and Treatment of Cancer; GTV, gross tumor volume; HD, Hodgkin disease; IFRT, involved-field radiation therapy; ILROG, International Lymphoma Radiation Oncology Group; INRT, involved-node radiation therapy; ISRT, involved-site radiation therapy; ITV, internal target volume; PTV, planning target volume.

The prepared five-question educational assessment is displayed in English in Figure 2. Question content was varied and assessed the topics of dose selection, differences between radiation design techniques such as IFRT and INRT, and trial data. The question format was multiple choice, and participants were provided identification numbers to track and pair their pre- and postintervention evaluations.

Nine students completed the pre- and postintervention assessment. The preintervention test was delivered before the 1-hour educational lecture and e-contouring session. Breakdown for the nine participants is provided in Table 1. The average score for the preintervention assessment was 75.6%. Scores ranged from 40% to 100%. Two participants had a score of 40%, and four participants answered all five questions correctly with a score of 100%. Question 2, which assessed dose selection, was the question that participants answered incorrectly the most, with five of nine participants answering it correctly. Questions 4 and 5, which assessed landmark trials and field design for lymphoma, respectively, were the questions answered correctly most often, with eight of nine participants answering these questions correctly.

**Table 1:**
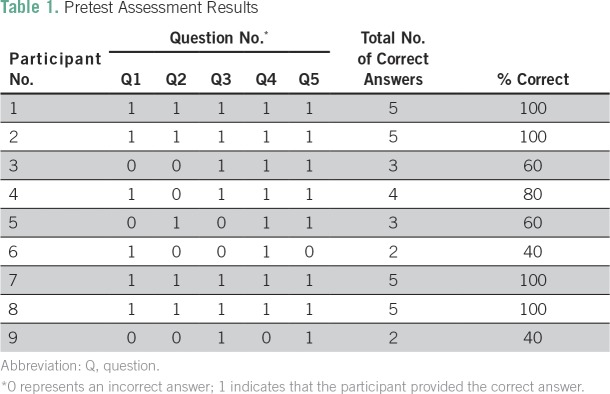
Pretest Assessment Results

After successful delivery of the educational lecture and e-contouring session, the postintervention assessment was delivered. Participants’ postintervention assessments were linked to their respective preintervention assessments by their given identifier, and results are listed in Table 2. The average score for the preintervention assessment was 86.7%. Scores ranged from 60% to 100%. One participant received a score of 60%, and four participants answered all questions correctly, with a score of 100%. Question 2, which assessed dose selection, remained the question that participants missed the most, with five of nine participants answering it correctly. Questions 1 and 4, which assessed the difference between ISRT and INRT and landmark trials, respectively, were the questions answered correctly most often, with nine of nine participants answering the questions correctly.

**Table 2:**
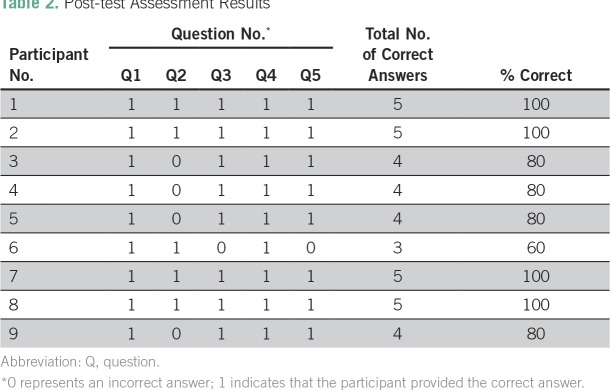
Post-test Assessment Results

Using a paired *t* test, a strong trend toward significance was detected between the preintervention and postintervention assessment scores (*P* = .051). No student had a decrease in score from the preintervention to postintervention evaluation. The topic with the lowest score for in both the preintervention and postintervention evaluations was dose selection as recommended by ILROG’s consensus guideline. The question with the greatest improvement in correct response was question 1, which improved from 67% correct responses on the preintervention test to 100% correct responses on the postintervention test.

## DISCUSSION

This represents, to our knowledge, the first international education intervention in Spanish incorporating e-contouring and using consensus guidelines with learning evaluation before and after intervention. As noted, the ILROG publications remain the most widely downloaded articles published by *International Journal of Radiation Oncology, Biology, and Physics*.^1,2,7^ Borne out of a shifting treatment paradigm in lymphoma, the guidelines present consensus recommendations on the contouring and treatment of both HD and NHL. With the evolution of planning spanning EFRT, IFRT, INRT, and ISRT, it is paramount that radiation oncologists remain informed and knowledgeable in their distinctions and design. INRT and ISRT, by design, incorporate the metabolic information provided by PET imaging. The use and access of PET in Latin America are expanding, with greater PET availability in the public health care system in Brazil as well as greater nationwide availability in Uruguay.^8,9^ Thus, disseminating up-to-date and evidence-based medical practices is a global issue, and not a domestic one, as more expensive imaging modalities like PET continue to spread and access to radiotherapy itself grows.^10^ As the world and, in particular, low- and middle-income countries (LMICs) continue to acquire newer machines and treatment modalities, it is a challenge for practitioners to not be outpaced by technologies.

In our intervention, we detected an almost statistically significant difference in scores before and after educational intervention (*P* = .051). For our intervention, we had nine completed pre- and postintervention assessments each, suggesting that with greater sample size it is probable a statistical difference would be detected. Our intervention was delivered in the target language, Spanish, of the audience. It was evidence based in that its material was rooted in the consensus guidelines established by ILROG. It also used e-contouring as a teaching supplement for participants to view three-dimensionally, including the contouring of targetstructures (gross tumor volume, clinical target volume, internal target volume, and planning target volume) and the organs at risk. Participants were also able to understand the distinctions in anatomy when supplementing simulation with a dose-reducing technique such as deep inspiratory breath hold.

There were limitations to our intervention. Given the technologic capabilities of the conference center, participants were not able to optimally use e-contouring to its fullest extent because each participant could not be provided with an individual computer or laptop because the interactive aspect of e-contouring software was not available for attendees. We acknowledge that it is suboptimal to impart practical knowledge without a hands-on e-contouring interaction. As has been stated, this experience served as a pilot intervention, and in that vein, the attendees, hosts, and educators all learned from the endeavor. We sought to complete this pilot to engage practitioners from LMICs in light of the fact that it is projected that, by 2035, more than two thirds of all cancer deaths will occur in LMICs. With the acquisition of more modern equipment and techniques in LMICs, practitioners are faced with the challenge of staying current on treatment planning with newer technology. Our work highlights the need to adapt e-contouring instruction to the resources available in LMICs. The e-contouring platform was only shown to the attendees as a didactic aid and not a user-facing platform available for attendees because ideal implementation was not feasible at the 2015 ALATRO meeting. Our pilot work demonstrates that a process is required to bring contouring courses to LMICs, even if the subject material is enabled for information and communication technology. Significant preparation must be laid out before the actual intervention because the contouring exercises take time and effort. This pilot served as a foray into Spanish-language instruction in an LMIC. Participants are now aware of an information and communication technology that can be considered in future ALATRO meetings to gain practical knowledge. As a result of this experience, R.B.M.V. has been invited to serve as a future e-contouring educator and plans to oversee e-contouring programs similar to the ones conducted at ESTRO and ASTRO. The exact purpose of the ASTRO e-contouring ambassador program is to work with staff to help host participants understand the logistics and technology needed to conduct an e-contouring session. What is reflected in our presented experience is that the hosts were most likely unaware of the resources needed for truly interactive participation, but with this experience, they can now be prepared.

Another limitation of the intervention is related to the time available for instruction. Given the time constraints, the educational assessment was limited to a five-question survey. Future research interventions would be improved by allowing individual access to the e-contouring cases, similar to what is provided at the American ASTRO e-contouring sessions. In addition, knowledge assessment would be strengthened not only by assessing participant knowledge content in a multiple-choice format, but also by using participant contouring itself as a metric of accuracy and understanding of didactic content. As the assessment demonstrated, future didactic sessions in management should further emphasize the importance of dose selection, particularly the how, when, and why.

Historical examples abound that underscore the value of appropriate contour and design within the field of radiation oncology. For the management of pancreatic cancer, the Radiation Therapy Oncology Group 97-04 trial evaluated the efficacy of continuous-infusion flourouracil versus gemcitabine before and after resection of pancreatic adenocarcinoma.^11^ A review of the trial results and the quality of radiation delivery defined the following two different groups: those that adhered per protocol to radiation design and those that did not.^12^ A statistically significant difference in median survival was detected favoring patients in the per-protocol cohort (log-rank *P* = .0077), and even in multivariable analysis adjusting for treatment arm, nodal involvement, tumor diameter, and margin status, radiation quality was significantly associated with survival (*P* = .014). A similar phenomenon of the effect of a lack of consensus in treatment field design was observed in the American College of Surgeons Oncology Group (ACOSOG) Z0011 trial, which evaluated the effect of axillary lymph node dissection on survival in women with invasive breast cancer with one to two positive sentinel lymph nodes. Trial results noted no significant difference in overall survival or disease-free survival (*P* = .25 and *P* = .14, respectively) based on arm assignment.^13^ However, review of the radiation field data of patients enrolled onto ACOSOG Z0011 with evaluable plans demonstrated that approximately 50% of patients across the trial received high tangent irradiation, which would provide more adequate coverage of the axilla.^14^ The outcomes suggest there may have been a possible influence of radiation design on the results of ACOSOG Z0011.

Together, the retrospective investigations examining ACOSOG Z0011 and the Radiation Therapy Oncology Group 97-04 trial stress the importance of radiation design quality and consistency. Radiation design is particularly important in lymphoma. The management of lymphoma across time has seen a shrinking in volumes targeted for definitive and consolidative management. Historically, large fields as seen in EFRT were used, resulting in the irradiation of substantial volumes of tissue. Over time, target volumes have shrunken through techniques such as IFRT, INRT, and ISRT, with the rationale of reducing toxicity by reducing irradiated volume. However, the risk of diminishing volumes is balanced by the increased chance of missing target, particularly because phase III randomized controlled trials do not exist comparing the different techniques in lymphoma management. Hence, accurate and evidence-based contouring and target delineation are vital for delivering optimal patient care.

Thus, we present a pilot strategy to deliver up-to-date and evidence-based content to radiation oncologists internationally. Although the change in pre- and postintervention assessments was not statistically significant as defined by *P* < .05, the *P* value in this pilot study (*P* = .051) with a sample size of nine completed assessments is reassuring and is most likely attributable to the study being underpowered. This strategy was a combination of both didactic lecture and e-contouring demonstration.

For many of the most common cancers in LMICs, radiation is an essential part of management.^10^ With an increasing gap in radiation oncology capacity and increasing incidence of cancer, there is increased demand not only for radiotherapy facilities and equipment, but also for properly educated health care professionals.^15,16^ Organizations such as ESTRO have a history of educational collaborations either within Europe or between Europe and other developed nations through programs such as the Fellowship in Anatomic Delineation and Contouring, which notably uses e-contouring through EduCase, and the Global Radiation Oncology Collaboration in Education.^17,18^ Apart from these collaborations between developed nations, e-contouring–based education interventions also exist in the developing world, with one such example being ASTRO’s EduCase online contouring breast modules that were incorporated into a pilot curriculum for the implementation of three-dimensional conformal breast radiation therapy in Armenia.^19^ We acknowledge that, in this pilot, we did not provide an ideal and interactive e-contouring experience for practical volume construction; however, we identified the obstacles and hurdles for improved future implementation for both the educating staff as well as the host site. Although contouring and structure delineation are key for optimal and safe radiotherapy delivery, also of importance is the correct dose selection and treatment rationale, for which we saw no improvement in score between pre- and postintervention assessments. It is possible that the 1-hour didactic time constraint limited the amount of information that could be easily synthesized. Future interventions may require more time with more emphasis placed on dose selection. Furthermore, to characterize why dose selection did not improve, future investigations should ask participants why the dose selection question is difficult. 

Although we used lymphoma as a sample pathology, the educational technique and basis are applicable to other cancers. Given the variability in execution in contouring and field design within radiation oncology, educational strategies are necessary as the specialty evolves and fields and modalities change to ensure optimal care and, in particular, to reduce the global inequity in cancer care.^15^ Such interventions recognize the art of medicine, and of radiation oncology in particular, but also recognize the evidence and data informing it.

## References

[1] Specht L, Yahalom J, Illidge T (2014). Modern radiation therapy for Hodgkin lymphoma: Field and dose guidelines from the International Lymphoma Radiation Oncology Group (ILROG). Int J Radiat Oncol Biol Phys.

[2] Illidge T, Specht L, Yahalom J (2014). Modern radiation therapy for nodal non-Hodgkin lymphoma-target definition and dose guidelines from the International Lymphoma Radiation Oncology Group. Int J Radiat Oncol Biol Phys.

[3] Reimann LC, Amendola B (2016). Highlights from the first International eCancer Conference on Oncology and Radiotherapy, 6-7 May 2016, Santiago, Chile. Ecancermedicalscience.

[4] Engert A, Plütschow A, Eich HT (2010). Reduced treatment intensity in patients with early-stage Hodgkin’s lymphoma. N Engl J Med.

[5] Lowry L, Smith P, Qian W (2011). Reduced dose radiotherapy for local control in non-Hodgkin lymphoma: A randomised phase III trial. Radiother Oncol.

[6] Hoskin PJ, Kirkwood AA, Popova B (2014). 4 Gy versus 24 Gy radiotherapy for patients with indolent lymphoma (FORT): A randomised phase 3 non-inferiority trial. Lancet Oncol.

[7] Yahalom J, Illidge T, Specht L (2015). Modern radiation therapy for extranodal lymphomas: Field and dose guidelines from the International Lymphoma Radiation Oncology Group. Int J Radiat Oncol Biol Phys.

[8] Strasser-Weippl K, Chavarri-Guerra Y, Villarreal-Garza C (2015). Progress and remaining challenges for cancer control in Latin America and the Caribbean. Lancet Oncol.

[9] Ministério da Saúde, Secretaria de Ciência, Tecnologia e Insumos Estratégicos: PET-CT no stadiamento e Avaliação da Resposta ao Tratamento dos Linfomas. http://conitec.gov.br/images/Relatorios/2015/Relatorio_PETLinfoma_FINAL.pdf

[10] Atun R, Jaffray DA, Barton MB (2015). Expanding global access to radiotherapy. Lancet Oncol.

[11] Regine WF, Winter KA, Abrams RA (2008). Fluorouracil vs gemcitabine chemotherapy before and after fluorouracil-based chemoradiation following resection of pancreatic adenocarcinoma: A randomized controlled trial. JAMA.

[12] Abrams RA, Winter KA, Regine WF (2012). Failure to adhere to protocol specified radiation therapy guidelines was associated with decreased survival in RTOG 9704: A phase III trial of adjuvant chemotherapy and chemoradiotherapy for patients with resected adenocarcinoma of the pancreas. Int J Radiat Oncol Biol Phys.

[13] Giuliano AE, Hunt KK, Ballman KV (2011). Axillary dissection vs no axillary dissection in women with invasive breast cancer and sentinel node metastasis: A randomized clinical trial. JAMA.

[14] Jagsi R, Chadha M, Moni J (2014). Radiation field design in the ACOSOG Z0011 (Alliance) trial. J Clin Oncol.

[15] Eriksen JG (2017). Postgraduate education in radiation oncology in low- and middle-income countries. Clin Oncol (R Coll Radiol).

[16] Grover S, Balogun OD, Yamoah K (2015). Training global oncologists: Addressing the global cancer control problem. Front Oncol.

[17] Eriksen JG, Salembier C, Rivera S (2014). Four years with FALCON: An ESTRO educational project—Achievements and perspectives. Radiother Oncol.

[18] Turner S, Eriksen JG, Trotter T (2015). Establishing a Global Radiation Oncology Collaboration in Education (GRaCE): Objectives and priorities. Radiother Oncol.

[19] Ngwa W, Ngoma T, Zietman A (2016). Closing the cancer divide through Ubuntu: Information and communication technology-powered models for global radiation oncology. Int J Radiat Oncol Biol Phys.

